# The NADPH Metabolic Network Regulates Human *αB-crystallin* Cardiomyopathy and Reductive Stress in *Drosophila melanogaster*

**DOI:** 10.1371/journal.pgen.1003544

**Published:** 2013-06-20

**Authors:** Heng B. Xie, Anthony Cammarato, Namakkal S. Rajasekaran, Huali Zhang, Jennifer A. Suggs, Ho-Chen Lin, Sanford I. Bernstein, Ivor J. Benjamin, Kent G. Golic

**Affiliations:** 1 Department of Biology, University of Utah, Salt Lake City, Utah, United States of America; 2 Department of Biology, San Diego State University, San Diego, California, United States of America; 3 Division of Cardiology, Department of Medicine, Johns Hopkins University, Baltimore, Maryland, United States of America; 4 Division of Cardiology, University of Utah School of Medicine, Salt Lake City, Utah, United States of America; 5 Department of Biochemistry, University of Utah School of Medicine, Salt Lake City, Utah, United States of America; Stanford University School of Medicine, United States of America

## Abstract

Dominant mutations in the *alpha-B crystallin* (*CryAB*) gene are responsible for a number of inherited human disorders, including cardiomyopathy, skeletal muscle myopathy, and cataracts. The cellular mechanisms of disease pathology for these disorders are not well understood. Among recent advances is that the disease state can be linked to a disturbance in the oxidation/reduction environment of the cell. In a mouse model, cardiomyopathy caused by the dominant *CryAB^R120G^* missense mutation was suppressed by mutation of the gene that encodes glucose 6-phosphate dehydrogenase (G6PD), one of the cell's primary sources of reducing equivalents in the form of NADPH. Here, we report the development of a *Drosophila* model for cellular dysfunction caused by this *CryAB* mutation. With this model, we confirmed the link between G6PD and mutant *CryAB* pathology by finding that reduction of G6PD expression suppressed the phenotype while overexpression enhanced it. Moreover, we find that expression of mutant CryAB in the *Drosophila* heart impaired cardiac function and increased heart tube dimensions, similar to the effects produced in mice and humans, and that reduction of G6PD ameliorated these effects. Finally, to determine whether CryAB pathology responds generally to NADPH levels we tested mutants or RNAi-mediated knockdowns of phosphogluconate dehydrogenase (PGD), isocitrate dehydrogenase (IDH), and malic enzyme (MEN), the other major enzymatic sources of NADPH, and we found that all are capable of suppressing CryAB^R120G^ pathology, confirming the link between NADP/H metabolism and CryAB.

## Author Summary

Cardiomyopathy is a specific form of heart disease that involves progressive restructuring of the heart muscle, resulting in reduced function and increased chance of sudden heart failure. Several mutations have been identified that cause inherited cardiomyopathy, including mutations in a gene called *alpha B-crystallin*. The work we report here puts this mutant gene into the fruit fly *Drosophila melanogaster*, where it causes heart defects that are similar to the human condition. Like cataracts reported in humans, this mutant gene also causes defective development of the fly eye. By examining defective fly hearts and eyes, and asking how genes involved in oxidation/reduction (redox) reactions influence these defects, we find that genes which control the redox environment of the cell also control the heart and eye defects. Oxidative stress, defined as an excess of harmful oxygen radicals, is a common concept in disease. Our work shows that in some circumstances, the genes that generate a more reduced cellular environment may also contribute to disease.

## Introduction

The maintenance and integrity of specialized functional structures such as sarcomeres, the basic unit of contractile force in striated muscles, are inextricably linked to the cellular machinery of molecular chaperones and protein quality control pathways. Evidence for this notion is provided by the identification of myopathic mutations in genes that encode proteins with chaperone function, such as *CryAB* and *Bag3*, and whose products have been localized to Z-discs. Moreover, an increasing number of genes encoding Z-disc associated proteins, such as desmin, ZASP, myotilin and filamin C are linked to myofibrillar diseases [1]. The Z-disc, which is formed by a complex network of diverse proteins, defines the structural boundaries of sarcomeres and integrates the actin filaments of neighboring contractile units. Major morphological and cellular hallmarks that define myofibrillar disorders include disintegration of the Z-disc lattice network, mitochondrial disruption, and ectopic protein aggregates.

The autosomal dominant *R120G* mutation in the *αB-crystallin* gene (*CryAB^R120G^*) manifests adult-onset cataracts, skeletal muscle weakness and heart failure [2]. CryAB, a small molecular weight heat shock protein, is expressed constitutively in the lens and in non-lenticular tissues associated with high rates of oxidative metabolism, such as heart and type I and type II skeletal muscle fibers. A primary function of CryAB in these tissues is to prevent aggregation of intermediate filament proteins such as desmin, a characteristic subcellular phenotype of desmin-related myopathies [3]. Earlier studies by several laboratories supported a loss-of-function mechanism for the *CryAB^R120G^* mutation, based on alterations of its secondary and quaternary structures, decreased interactions for client substrates with intermediate filaments and reduced stability on heat denaturation *in vitro* [4], [5]. Subsequently, expression of either the mouse or human *CryAB^R120G^* allele in the mouse heart produced cardiomyopathy, heart failure and shortened lifespan, all of which phenocopy the disease condition in humans [6], [7]. Additional missense, truncation and autosomal recessive mutations have underscored the dominant inheritance patterns of disease-causing CryAB expression but their underlying molecular mechanism(s) have remained elusive.

An important concept in the pathogenesis of neurodegenerative and, perhaps, myofibrillar disease is that misfolding and aggregation of destabilized mutant proteins trigger ‘toxic’ gain of function etiologies [8]. It has been hypothesized that the accumulation of amyloidogenic aggregates is preceded by the appearance of soluble, oligomeric species whose toxicities arise from multiple, non-exclusive mechanism(s) mediated by (1) flexible hydrophobic surfaces that promote aberrant interactions and sequestration [9], (2) poor clearance mechanisms that disrupt central protein quality control and propagate folding defects [10], [11], and (3) perhaps by compromised lipid integrity as suggested by *in vitro* model membrane systems [12]. Although *CryAB^R120G^* does not appear to result from a “classical” amyloid, recent studies have shown that aggregates of mutant CryAB are recognized by the anti-oligomer A11 [13], [14] providing a link with well-known amyloid protein aggregation diseases such as Alzheimer's and Huntington's diseases.

Our recent work on a mouse model of the inherited human *CryAB^R120G^* cardiomyopathy has provided the first persuasive case for pathology resulting from “reductive”, as opposed to oxidative, stress in disease pathogenesis. The molecular events and damaging effects of reactive oxygen species (ROS) on biological molecules and systems are well known. When ROS are generated in excess of a cell's capacity to neutralize them, by enzymatic and non-enzymatic antioxidant pathways, the cell experiences oxidative stress. A large number of disease states have been attributed, in whole or in part, to damage from ROS or oxidative stress. However, there has been little appreciation for the possibility that an excess of reducing equivalents might also cause problems for the cell. Reductive stress can be induced by supplying strong reducing compounds, such as dithiothreitol, to cells in culture, but only recently has it been shown by Rajasekaran and coworkers that reductive stress may occur as a pathological state consequent to expression of a mutant protein. *CryAB^R120G^*-induced cardiomyopathy was accompanied by a significant shift towards a more reduced intracellular environment, as measured by the glutathione redox couple [7]. The dominance of *CryAB^R120G^*, and its unexpected link to an excessively reduced environment, suggested a ‘toxic’ gain of function mechanism.

To investigate the reductive stress hypothesis of CryAB^R120G^ pathology, we have developed the first *Drosophila melanogaster* model of human *CryAB^R120G^* toxicity in multiple organs and tissues. Because there is a strong evolutionary conservation of key developmental and metabolic pathways between humans and *Drosophila* [15]–[18], studies of human conditions in *Drosophila* have been very productive. This has been especially true in the fields of heritable developmental defects, including congenital heart diseases, aging-related conditions and neurodegenerative diseases [19]–[22]. Recently, with advanced microscopic technology [23], *Drosophila* has been used to model human cardiac physiology and aging [24]–[27]. Interestingly, flies with a dilated heart have been reported in certain genetic backgrounds [28]. Strikingly, cardiac dilation is observed in patients with protein aggregation cardiomyopathy and in mice that over-express *CryAB^R120G^* [7].

In this study, we demonstrate that *CryAB^R120G^* pathologies in the fly heart and eye are regulated by key enzymes that reduce nicotinamide adenine dinucleotide phosphate (NADP) to NADPH. As was the case in the mouse heart, the deleterious effects of expressing *CryAB^R120G^* were strongly ameliorated by knockdown or mutation of the gene encoding G6PD. We further exploited the fly model and report that reduced function of other major generators of NADPH also strongly suppressed the *CryAB^R120G^* phenotype, implicating the entire cellular NADP/NADPH network in *CryAB^R120G^* pathology.

## Results

### *Drosophila* heart dysfunction caused by human CryAB^R120G^

To extend our tests of the reductive stress hypothesis of CyrAB^R120G^ pathology, we used the *Gal4-UAS* modular expression system [29], [30] to permit expression of the human *CryAB^R120G^* allele in various cell types. We first generated transformants carrying either a wild-type *UAS-CryAB^+^* or *UAS-CryAB^R120G^* construct. Next, to determine whether *CryAB^R120G^* expression would affect cardiac function in *Drosophila*, we drove its expression in the fly heart with a *Hand-Gal4* driver. We confirmed cardiac-specific expression from *Hand-Gal4* by examining GFP fluorescence using a *UAS*-*CryAB^R120G^-GFP* fusion construct. Visual inspection of fluorescent micrographs confirmed cardiomyocyte restricted expression of the construct. Remarkably, the marked CryAB^R120G^ appeared targeted to a repetitive myofibrillar component of the cardiac fibers of flies, as found in higher organisms [31], likely the Z-discs (Figure S1).

**Figure 1:**
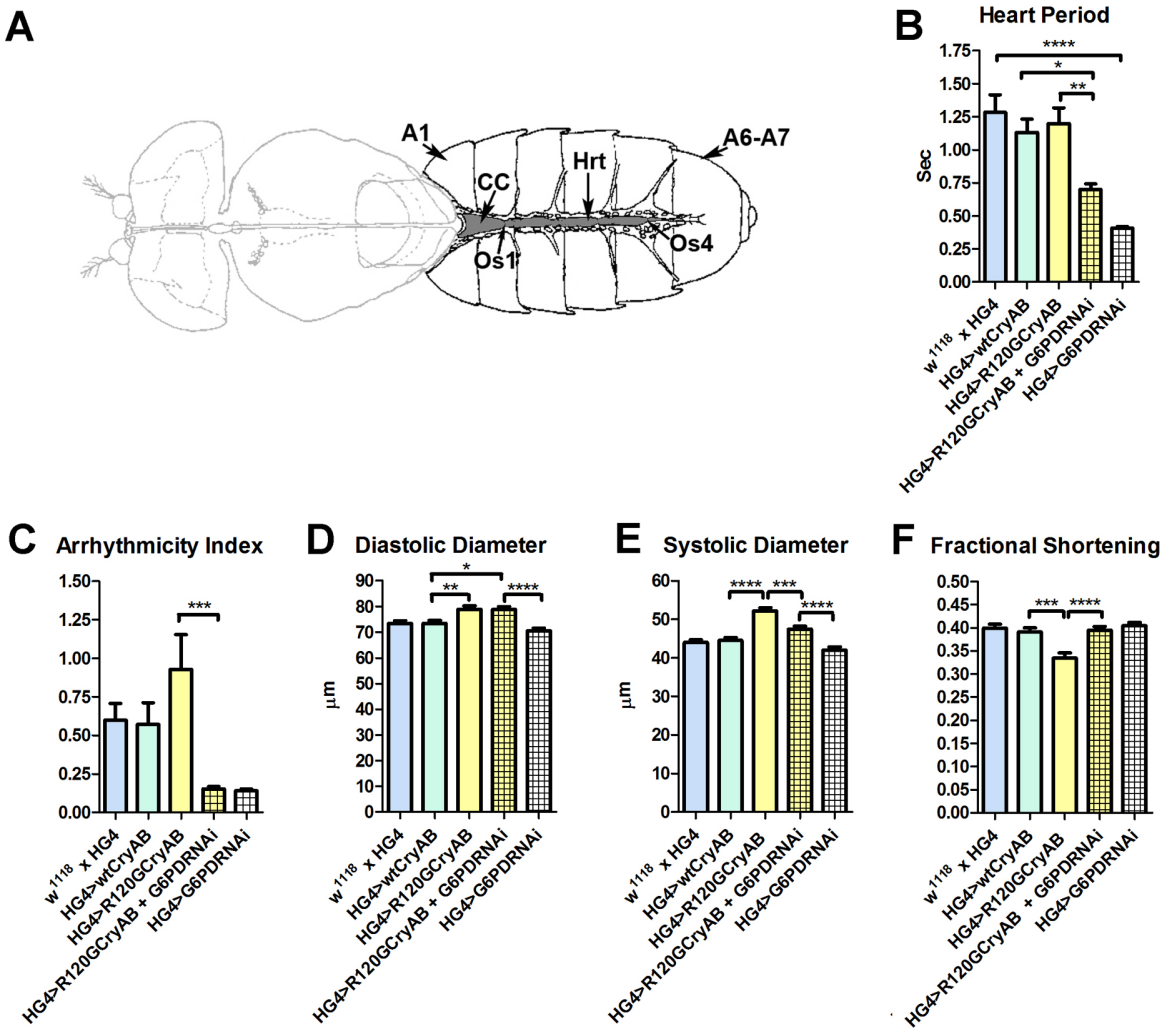
The *Drosophila* heart and physiological analysis. (A) The abdominally located *Drosophila* heart (Hrt) is a simple linear tube that is divided into an anterior, conical chamber (CC) and three posterior compartments. A1 = abdominal segment 1; A6–A7 = abdominal segments 6 and 7; Os = ostia inflow tracts (modified from [66]). (B–F) Cardiac-restricted expression of wild type human *CryAB* (HG4>wtCryAB) had no significant effect on any measured parameter of cardiac performance in *Drosophila*. Several indices of cardiac function were influenced by the expression of mutant *CryAB^R120G^* (HG4>R120GCryAB). The diameter across the heart tubes during (D) diastole and (E) systole were significantly greater, and (F) percent fractional shortening significantly lower relative to HG4>wtCryAB flies. Knockdown of G6PD in *CryAB^R120G^* mutant hearts (HG4>R120GCryAB+G6PDRNAi) substantially improved cardiac performance of the mutant hearts as evidenced by (B) significant decreases in heart period, (C) arrhythmic beating patterns, (E) systolic dimensions and (F) significantly increased fractional shortening relative to HG4>R120GCryAB hearts. * P≤0.05, ** P≤0.01, *** P≤0.001, **** P≤0.0001.

To investigate the effects of human wild type or mutant *CryAB* expression on the simple, linear *Drosophila* cardiac tube (Figure 1A) we imaged surgically exposed beating hearts and tracked wall movements of semi-intact flies using direct immersion DIC optics in conjunction with a high speed digital video camera [32]. We characterized the effects of CryAB^+^ and CryAB^R120G^ on the contractile performance and general morphology of *Drosophila* hearts.

Heart period, which is defined as the length of time between the ends of two consecutive diastolic intervals, and arrhythmia indices, a quantitative measure that reflects cardiac rhythmicity and permits exploration of heart rhythm irregularities, were calculated for ∼45 three week old semi-intact *Drosophila* from each line. Cardiac diameters were measured directly from individual video frames at peak diastolic and systolic time points at multiple locations along the linear portion of abdominal segment three of each heart tube. These measurements revealed that expression human *CryAB^+^* did not significantly perturb any analyzed index of cardiac function relative to control hearts (Figure 1, Figure S2). In contrast, expression of *CryAB^R120G^* significantly affected several parameters of heart function. Arrhythmic beating patterns appeared to increase (Figure 1C), although these trends were not statistically significant. Diastolic and systolic diameters were significantly increased in response to *CryAB^R120G^* expression, and fractional shortening of the fly heart was significantly reduced thus notably impairing *Drosophila* cardiac function (Figure 1D–F). In accord with the mouse observations [7], simultaneous RNAi-mediated knockdown of G6PD significantly improved several indices of cardiac function and overall heart performance. Hearts expressing both *CryAB^R120G^* and RNAi targeted against *Zw* (the gene encoding G6PD) exhibited significantly shorter heart periods (increased heart rates; Figure 1B), significantly reduced arrhythmia indices and systolic diameters, and significantly greater fractional shortening (Figure 1C–F). Interestingly, cardiac restricted expression of G6PD RNAi alone significantly decreased heart periods relative to those of *w^1118^* x *HandGal4* control hearts (Figure 1B).

**Figure 2:**
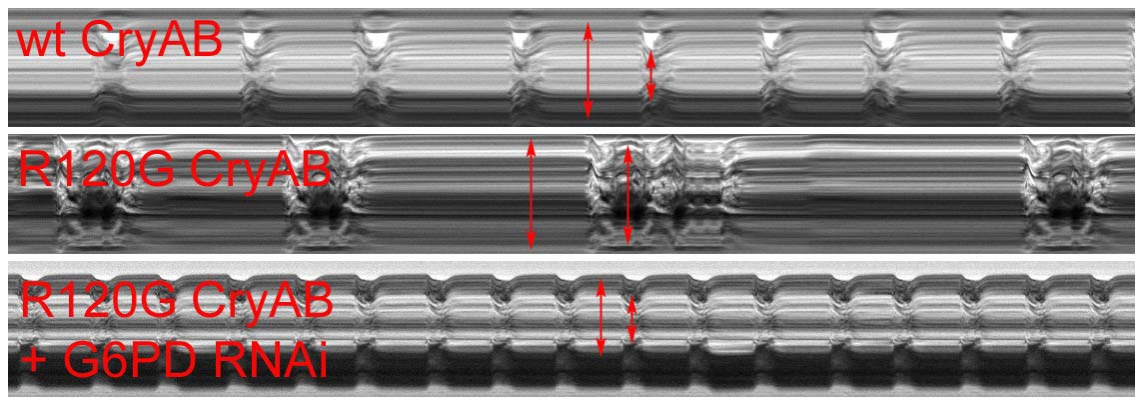
M-modes of heart tubes expressing control and mutant CryAB. M-mode traces reveal the positions of the *Drosophila* heart wall edges (Y direction) over time (X direction). M-modes from hearts expressing wild type CryAB exhibit fairly regular contraction and relaxation cycles. However, movement traces of cardiac tubes expressing mutant CryAB^R120G^ reveal hearts with dilated dimensions, a lower extent of shortening and arrhythmic beating patterns relative to controls. M-modes from hearts expressing mutant *CryAB^R120G^* and *Zw* (G6PD) RNAi suggest that knockdown of G6PD increases heart rate, promotes rhythmic beating patterns and rescues the cardiac tube dimensions of the mutant hearts.

Many of these differences in contracting heart tubes were qualitatively visualized via M-mode traces, which display the dynamics of cardiac contractions of representative hearts from the various genotypes (Figure 2). These traces show the positions of the heart wall edges (Y direction) over time (X direction). M-modes from semi-intact heart preparations from control flies show fairly regular contractions. However, these traces reveal a subtly arrhythmic beating pattern in *CryAB^R120G^* cardiac tubes relative to controls. Further, the *CryAB^R120G^* hearts were dilated, and exhibited a lower extent of shortening. Co-expression of *Zw* RNAi increased heart rate, promoted rhythmic beating and rescued percent fractional shortening in the *CryAB^R120G^* mutant hearts. Thus, overall, cardiac output of the mutant hearts is likely to be significantly enhanced by reducing the enzymatic activity of G6PD in flies as found in mouse models [7].

### CryAB^R120G^-induced defects in non-heart tissues

We also asked whether *CryAB* expression produced any deleterious effects if expressed in other tissues. When *CryAB^+^* was expressed ubiquitously (using a *Tub-Gal4* driver), or in the eye only (using either *ey-Gal4* or *GMR-Gal4* drivers) no abnormal phenotypes were observed in 32 of 33 lines, in spite of easily detectable expression (in eight lines examined by Western blotting; not shown). We did recover a single *CryAB^+^* transformant that produced a rough eye phenotype. However, the insertion was located upstream of the *escargot* (*esg*) gene, a location where mis-expression elements are known to produce rough eyes by promoting *esg* expression [33]. We used qRT-PCR and verified that this particular *CryAB^+^* insertion also drove overexpression of *esg*. We therefore attribute its phenotype to *esg* overexpression, and not to *CryAB* ^+^.

**Figure 3:**
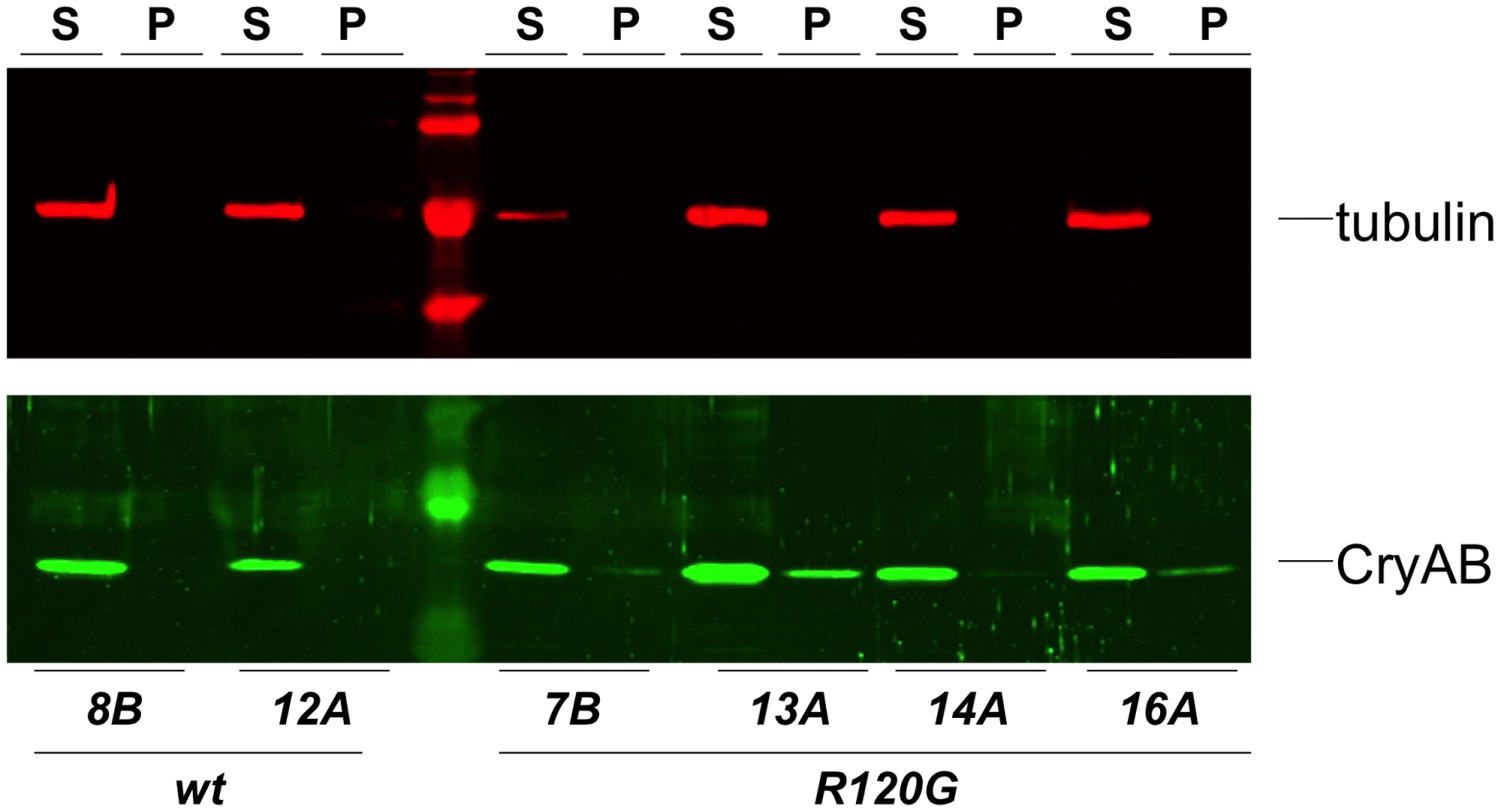
Solubility of CryAB in the fly eye. Western blots of proteins extracted from *Drosophila* heads that expressed either wildtype (wt) or mutant (R120G) CryAB, to produce soluble (S) and insoluble (P) fractions. The top panel shows the same blot probed with an anti-tubulin antibody as a control; the bottom panel shows the blot probed with an anti-CryAB antibody.

In contrast, we identified four *UAS-CryAB^R120G^* lines that produced complete or partial lethality with ubiquitous expression (using either *A5C-Gal4* or *Tub-Gal4* drivers). Western blotting of protein extracts from the single line with any survivors revealed abundant *CryAB^R120G^* expression. No expression was detected in seven fully viable lines (Figure S3). We speculate that the high frequency of non-expressing transformant lines reflects a strong selection against lines that show even slightly leaky expression. Subsequent Western blots of flies with expression limited to the eye (which allows survival) revealed that the three lines that were lethal when constitutively expressed also had abundant *Gal4*-induced expression of *CryAB^R120G^* (Figure 3).

**Figure 4:**
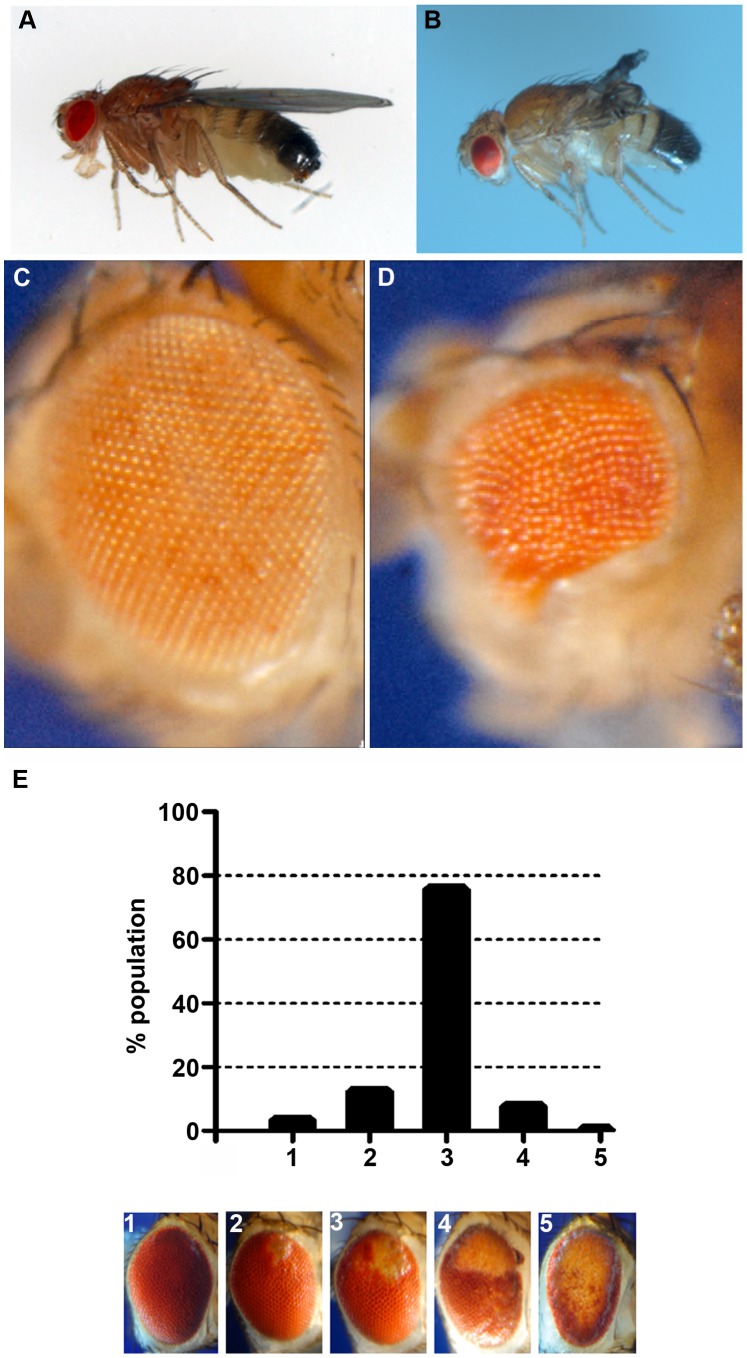
Phenotypes produced by *CryAB^R120G^* expression. Examples of wild-type (CryAB^+^) and CryAB^R120G^ phenotypes, respectively, are shown for expression in the wing (A, B) and expression in the undifferentiated eye disc driven by *eyGal4* (C, D). When *CryAB^R120G^* expression is driven late in eye development with *GMR-Gal4* a strong phenotype is produced with somewhat variable expressivity. The distribution of phenotypes, and corresponding images, are shown in E. The *CryAB^R120G^* line 16A was used for these tests.

When *CryAB^R120G^* expression was driven in the wing, flies with deformed and mis-shapen wings were produced (Figure 4B), while expression in the eye throughout development (with *ey-Gal4*) resulted in variably rough and small eyes (Figure 4D). A stronger and more consistent eye phenotype was observed when expression was driven in differentiating eye discs (with *GMR-Gal4*), characterized by irregular patterning and loss of ommatidia and pigment (Figure 4E).

Cardiomyopathy caused by mutations in *CryAB* is correlated with the presence of cytoplasmic protein aggregates containing CryAB and a number of other proteins [7], [14]. To determine whether human CryAB^R120G^ in *Drosophila* was also found in aggregates we extracted Triton-X 100 soluble and insoluble protein fractions from eyes expressing *CryAB^+^* and *CryAB^R120G^* and used Western blotting to determine the distribution of CryAB between these fractions (Figure 3). We found a portion of the CryAB^R120G^ mutant protein in the insoluble pellet fraction (6.6%±2.9, 23%±13, 1.9%±1.0 and 4.9%±1.0 for lines 7B, 13A, 14A and 16A, respectively), while CryAB^+^ protein was found virtually exclusively in the soluble fraction (with only 0.3%±0.2% and 0.2%±0.1% in the pellet for lines 8B and 12A, respectively). Thus, CryAB^R120G^ in *Drosophila* shares the characteristic of being found, in part, in insoluble aggregates.

The eye is dispensable for normal development and viability, and is an established model for examining the cellular effects of human neurodegenerative disease genes [34], [35]. Therefore, we chose to use the phenotype produced by *GMR-Gal4 UAS-CryAB^R120G^* in the eye as a model to test the reductive stress hypothesis. Eye phenotypes were quantitated by scoring into five groups, ranging from ∼wildtype (category 1), to having all or nearly all of the eye strongly affected (category 5; Figure 4E).

**Figure 5:**
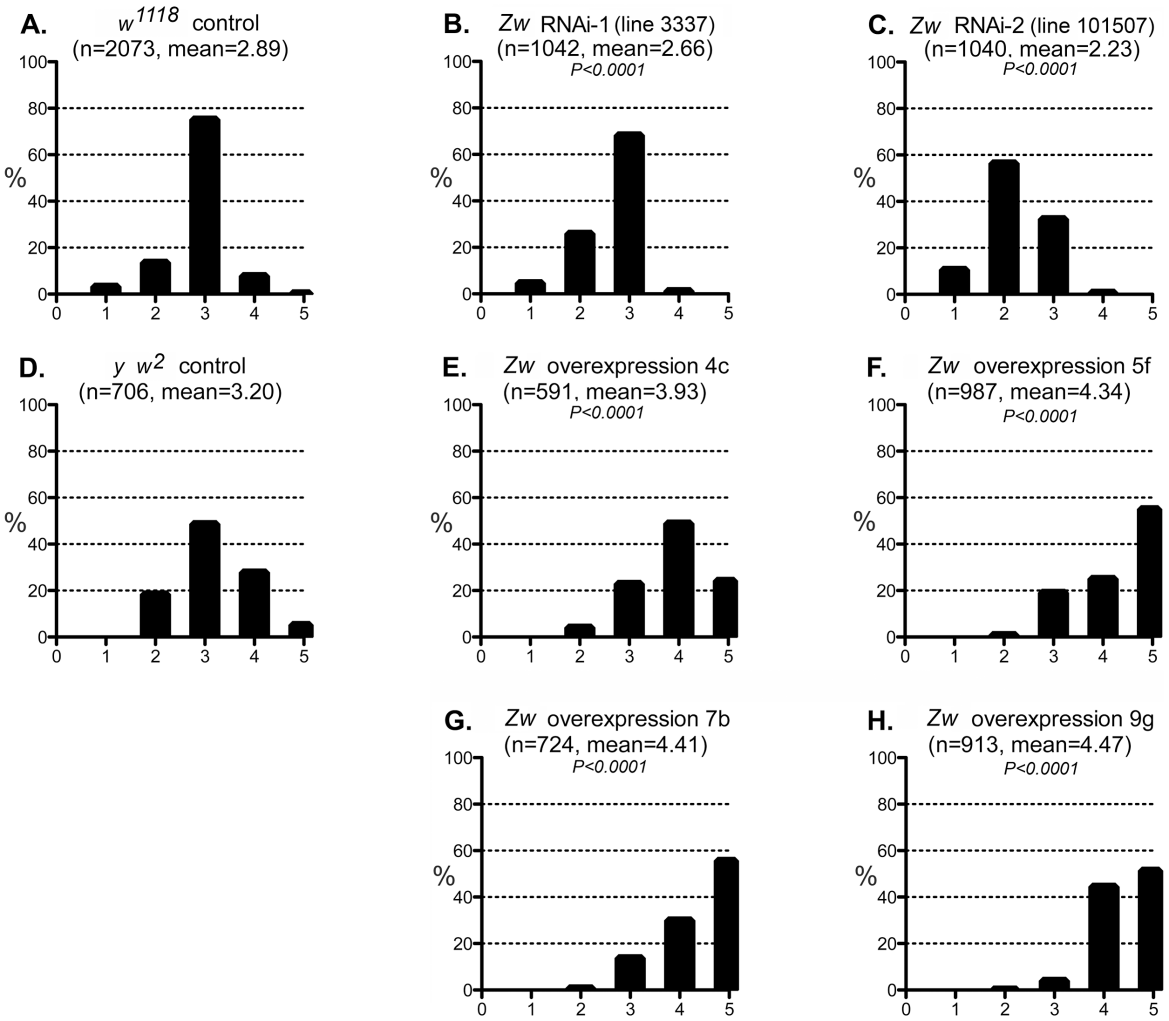
Effect of G6PD variation on the CryAB^R120G^ phenotype. A–C: The phenotypic distribution produced by *GMR>CryAB^R120G^* in a *w^1118^* control strain background (A), and the altered distributions produced by RNAi-mediated knockdown of *Zw* (B, C). D-H: The CryAB^R120G^ phenotypic distribution in a *y w^2^* control background (D) compared to phenotypes when *Zw* is overexpressed (E–H). The number of eyes scored is given as n; *P* is given for comparison with the appropriate control (A for B, C; D for E–H). Controls were chosen to most closely match the genetic background of the test strains. The *CryAB^R120G^* line 16A was used for these tests.

We then tested whether a reduction of G6PD could suppress the CryAB^R120G^ phenotype, as it does in mouse and fly hearts. Two RNAi lines that significantly reduced expression of *Zw* both suppressed the CryAB^R120G^ eye phenotype (Figure 5B,C; Table S1). To extend these observations we then asked whether overexpression of *Zw* would exacerbate the CryAB^R120G^ phenotype. Four overexpression lines were tested and, unlike the heart phenotype (Figure S2), all strongly enhanced the CryAB^R120G^ phenotype (Figure 5E–H; Table S1). The results shown in Figure 5 using the *CryAB^R120G^* line *16A* were confirmed with the independent *CryAB^R120G^* insertion line *14A* that also has a strong eye phenotype: responses to *Zw* knockdown and overexpression were extremely similar for the two lines (not shown). There was no phenotypic effect of altered G6PD levels on normal eyes.

We considered the possibility that the *UAS*-controlled RNAi constructs might titrate GAL4 and produce apparent suppression simply through reduced expression of *CryAB^R120G^*. However, since the G6PD overexpression constructs are also driven by GAL4, but they enhance the CryAB^R120G^ phenotype, this concern appears unfounded.

**Figure 6:**
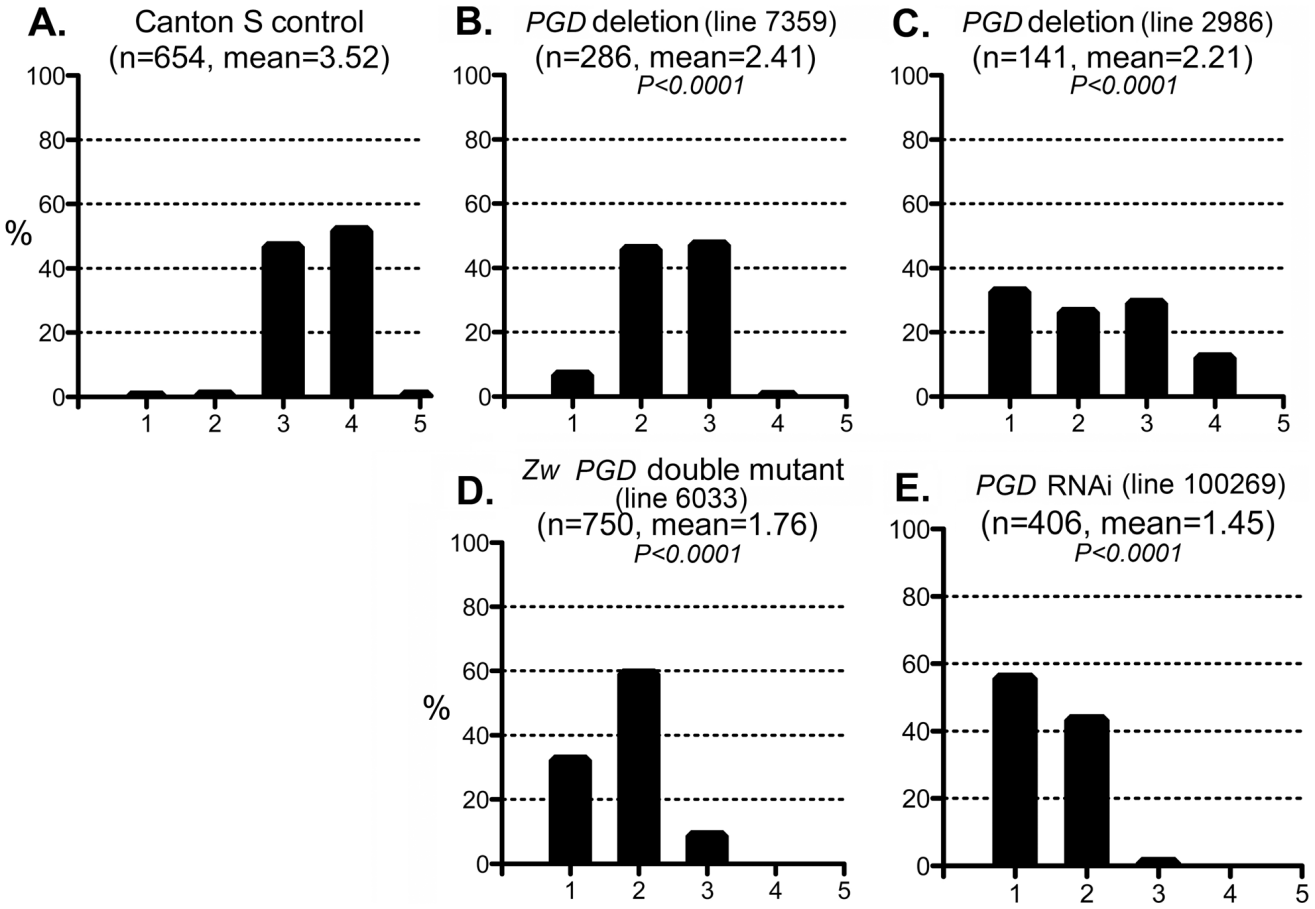
Effect of PGD reduction on the CryAB^R120G^ phenotype. The phenotypic distribution in the Canton S control (A) compared to that of deletions that remove *Pgd* (B, C), a *Zw Pgd* double mutant (D), and RNAi-mediated knockdown of *Pgd* (E). The *CryAB^R120G^* line 16A was used for these tests. In B–D, heterozygous +/− females were assayed.

The enzyme 6-phosphogluconate dehydrogenase (PGD) acts downstream of G6PD in the pentose phosphate pathway, and like G6PD, reduces NADP to NADPH. We tested whether mutations in the gene encoding PGD (*Pgd*) would also affect the CryAB^R120G^ phenotype. Two deletions that removed the gene (along with neighboring genes) suppressed the CryAB^R120G^ phenotype when tested as heterozygotes (Figure 6B,C; Table S1). In addition, flies carrying a chromosome with mutations in *Zw* and *Pgd*, in heterozygous condition, also showed strong suppression of the CryAB^R120G^ phenotype (Figure 6D). Finally, RNAi directed against *Pgd* produced very strong suppression of the CryAB^R120G^ phenotype (Figure 6E; Table S1). These mutants or RNAi knockdowns, by themselves, had no effect on the phenotype of normal eyes.

**Figure 7:**
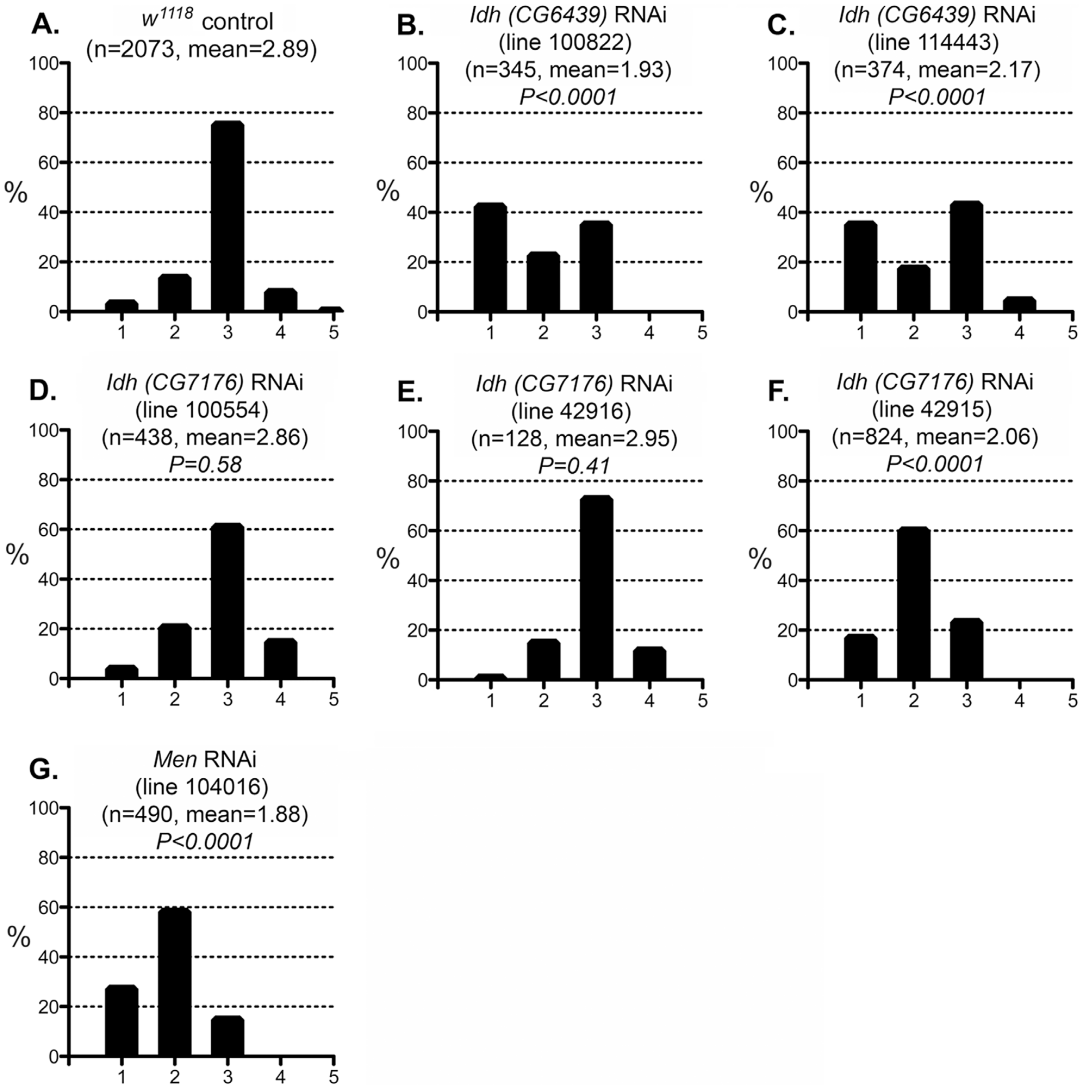
Effect of IDH and MEN reduction on the CryAB^R120G^ phenotype. The phenotypic distribution in the *w^1118^* control (A) compared to RNAi-mediated knockdown of the putative mitochondrial IDH (B, C) or cytoplasmic IDH (D–F), or MEN (G). The *CryAB^R120G^* line 16A was used for these tests.

These results are consistent with the reductive stress hypothesis, but it is still conceivable that G6PD and PGD influence CryAB^R120G^ pathology via other products of the pentose phosphate pathway. To determine whether varying the enzymatic production of NADPH would affect the *CryAB^R120G^* phenotype, regardless of the source of that variation, we tested whether alteration of isocitrate dehydrogenase (IDH) or malic enzyme (MEN) levels would affect the phenotype. We observed that RNAi-mediated knockdown of IDH (either the putative mitochondrial (*CG6439*) or cytoplasmic (*CG7176*) form) was capable of suppressing the *CryAB^R120G^* phenotype (Figure 7B–F; Table S1). Knockdown of MEN also suppressed the *CryAB^R120G^* phenotype (Figure 7G; Table S1). Knockdown of IDH or MEN had no effect on normal eyes. Although only one of three lines expressing RNAi against *Idh* (*CG7176*) achieved significant suppression of the *CryAB^R120G^* phenotype, it is also the case that these lines only achieved an ∼20–30% reduction in *Idh* RNA levels. In the cases of *Zw, Pgd* and *Men*, where strong suppression was observed, ∼40–50% knockdowns of RNA levels were achieved (Table S1).

**Figure 8:**
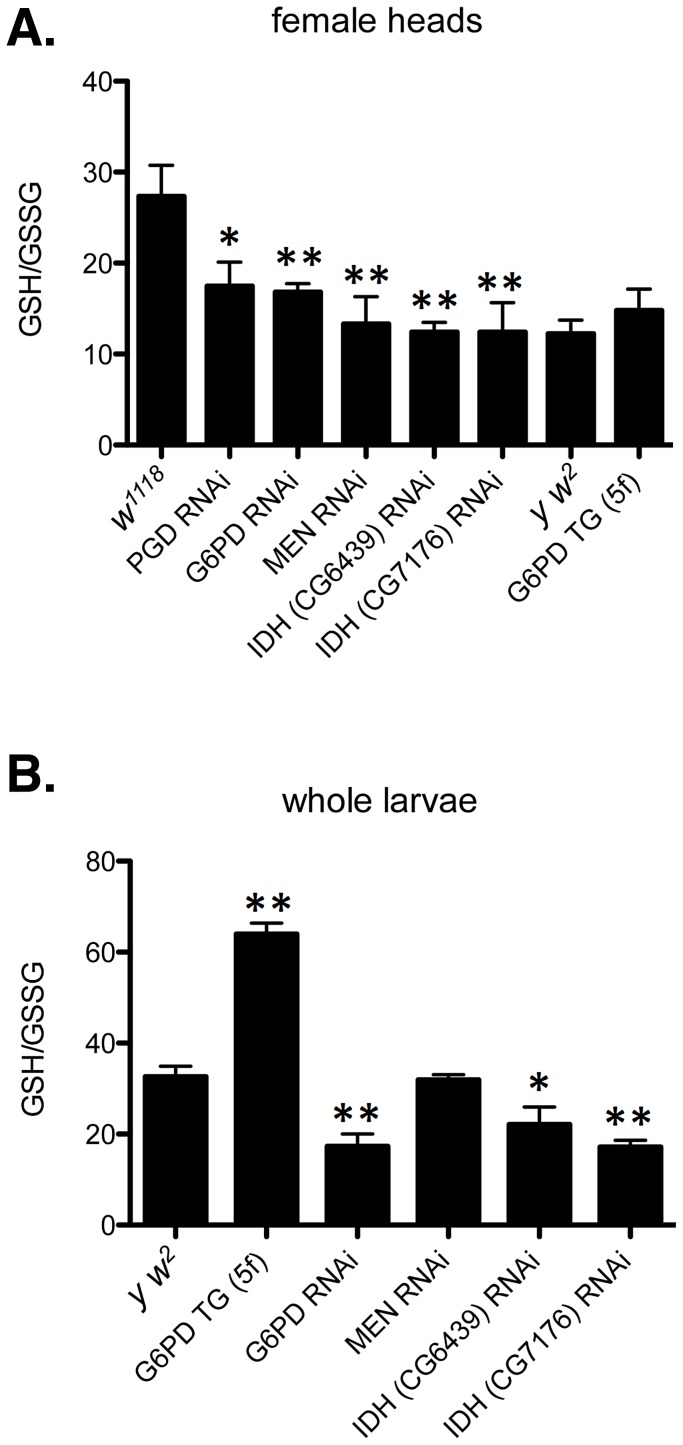
Redox environment is altered by changing the effective dosage of genes involved in NADP/H metabolism. (A) The GSH∶GSSG ratio was measured in the heads of female flies with RNAi-mediated knockdown of PGD, G6PD, MEN, or IDH, or overexpression of G6PD (G6PD TG). (B) GSH and GSSG were also measured in whole larvae expressing RNAi or extra transgenic copies under the control of tubulin-Gal4. All data points represent a set of at least 3 independent replicas, and are reported here as the mean ratio of nmol GSH to nmol GSSG (calculated as GSH equivalents) ± SE. Statistical results are for comparison with *w^1118^* for knockdowns in A; *y w^2^* for G6PD overexpression in A; and *y w^2^* for all comparisons in B. (** P<0.05, * P<0.1).

To assess the redox environment of cells in response to altered dosage of genes that mediate NADP/H metabolism we carried out two series of experiments to determine the ratio of reduced to oxidized glutathione (GSH∶GSSG). When RNAi-mediated knockdown of G6PD, PGD, MEN or IDH was driven in heads with *GMR-Gal4* the GSH∶GSSG ratio was reduced in every case (Figure 8A), though the reduction as a result of PGD knockdown was not significant at *P* = 0.05 level. The results confirm our supposition that these knockdowns impair cells' ability to generate NADPH. We observed a slight, though not statistically significant, increase in the GSH∶GSSG ratio when G6PD was overexpressed in heads. However, when we assayed whole larvae, with expression driven ubiquitously, G6PD overexpression produced a very large and significant increase in the GSH∶GSSG ratio (Figure 8B). These results confirm the involvement of the NADP/H network in cellular dysfunction produced by *CryAB^R120G^* expression and strongly implicate reductive stress as the causative agent for pathology.

One concern that arose when assessing the phenotypic effect of human *CryAB^R120G^* expression was that two *Drosophila* lines (*7B* and *13A*) showed only mild phenotypic effects in the eye (Table S2), despite exhibiting robust protein expression (Figure 3). However, these two lines do show rough eye phenotypes if subjected to a strong heat shock during late larval/early pupal development, a treatment that also strongly enhances the phenotype of lines *14A* and *16A* (Table S2). Flies carrying *GMR-Gal4* alone showed no eye phenotype when subjected to this heat shock. Moreover, one of these lines (7B) also exhibits a wing phenotype when expression is driven in the wing (Table S2).

To test whether these particular insertions might be associated with suppression of the *CryAB^R120G^* phenotype, we combined them with *GMR-Gal4* and the *CryAB^R120G^* line *16A* that was used in the experiments of Figures 4, 5 and 7. The combination of line *7B* or *13A* with *16A* resulted in an eye phenotype that was reduced from that of *16A* alone, indicating that these two lines do suppress the *CryAB^R120G^* eye phenotype to some degree (Figure S4). We have not determined why these two lines show little phenotypic consequence in the eye, but we speculate that it may have to do with subtle differences in timing of gene expression. Perhaps slightly earlier onset of expression in lines *7B* or *13A* results in a protective response, in the same way that mild heat shocks, by inducing synthesis of heat shock proteins, can protect against subsequent, more severe heat shocks [36], [37]. Alternatively, the expression of genes in the region of the *7B* and *13A* insertion loci might be altered to result in phenotypic suppression. In spite of these lines showing only mild effects, our experiments convincingly show that *CryAB^R120G^* expression, but not *CryAB^+^*, can cause strong phenotypic effects in the fly heart, eyes and wings. Moreover, detailed examination of the heart and wing defects show that they are responsive to altered levels of the enzymes that reduce NADP to NADPH.

## Discussion

Our results show that *Drosophila* provides a suitable model in which to study the pathology of the human *CryAB^R120G^* mutation. Expression of the mutant allele, but not the wildtype, in fly hearts, causes heart dilation and dysfunction very reminiscent of the cardiomyopathy produced in humans carrying this dominant allele [2]. We also found that a reduced level of G6PD ameliorates many of the perturbed cardiac functional parameters in *CryAB^R120G^* flies, just as it does in the *CryAB^R120G^* mouse [7]. Despite a lack of reduction in *CryAB^R120G^* diastolic diameters in response to *Zw* (G6PD) RNAi co-expression, systolic diameters in the double mutants were rescued and did not significantly differ from those found in fly hearts expressing wildtype human *CryAB*. Thus, fractional shortening was indistinguishable between wildtype *CryAB*-expressing and *CryAB^R120G^*+*Zw* RNAi-expressing hearts. Furthermore, cardiac restricted expression of *Zw* RNAi, either with *CryAB^R120G^* or alone, significantly increased heart rates relative to control hearts. This suggests *G6PD* deficiency can improve cardiac output in either mutant or non-mutant backgrounds and may be a potent modifier of cardiac function.

In *Drosophila*, overexpression of G6PD can extend lifespan and protect against oxidative stress [38]. In mammalian cells, overexpression of wildtype small heat shock proteins leads to increased G6PD expression and protection against oxidative stress [39]. Our finding, that reduction of G6PD can be beneficial in some circumstances, is also not without precedent. Some studies have suggested a link between *G6PD* deficiency and protection against cardiovascular disease in humans, although such findings have not been replicated in larger patient studies [40]–[42]. Previous work from one of our laboratories showed that G6PD reduction is highly beneficial in one specific case — when human *CryAB^R120G^* is expressed in the mouse heart [7]. We do not see a conflict in these differing outcomes, but instead conclude that the effect of modifying G6PD levels may range from beneficial to deleterious, with the outcome determined by the constellation of genetic variation present in individuals' genomes and the environmental stressors that they experience.

To generate an experimental paradigm suited to rapid genetic exploration we expressed *CryAB^R120G^* in the fly eye and found that it strongly disturbs normal development and pattern formation. The eye phenotype was also responsive to altered G6PD levels, validating it as a model for investigation of the underlying mechanism of *CryAB^R120G^* pathology. Unlike a recent report [43], we saw no abnormal eye phenotype that could be attributed to expression of the wild-type human *CryAB* gene. In the one case where we did observe an eye phenotype it most likely resulted from induced expression of the *esg* gene that lay adjacent to that particular insertion of the *CryAB* transgene. Our findings indicate that *CryAB^R120G^* induces cellular dysfunction in both the heart and the eye, or lethality if expressed ubiquitously, while wild-type *CryAB* is relatively benign.

The human disease produced by the *CryAB^R120G^* allele is sometimes called Desmin-Related Myopathy (DRM), owing to the presence of desmin in the characteristic cytoplasmic protein aggregates, and to similarities with diseases caused by mutations in the gene encoding the intermediate filament desmin [2], [6], [44]. Although *Drosophila* do not have a desmin homolog, several lines of evidence argue that the cellular dysfunction in *Drosophila* resulting from *CryAB^R120G^* expression is, nonetheless, a legitimate model for this disease. First, a number of other proteins have been identified within the aggregates, including (at least) another small heat shock protein and G6PD [7], [14], both of which have homologs in *Drosophila*. Second, CryAB^R120G^ causes the formation of cytoplasmic aggregates even when expressed in human cell types that do not express desmin [45], [46], and our results show that a portion of the CryAB^R120G^ is also found in aggregates in *Drosophila*. Third, the identical response of mouse and *Drosophila CryAB^R120G^* pathologies to G6PD reduction strongly suggests an identical mechanism of action.

**Figure 9:**
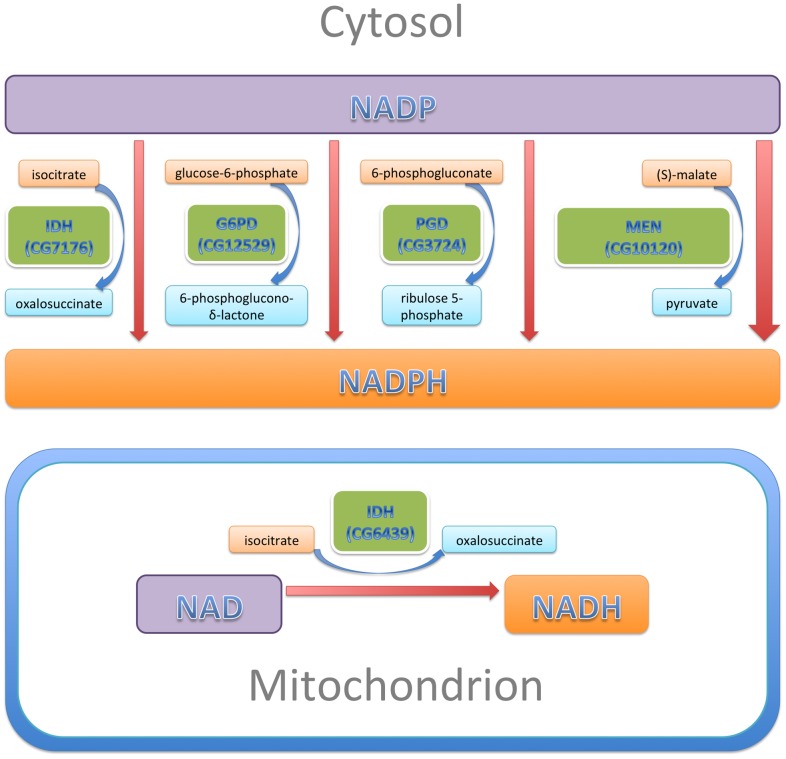
NADP/H metabolism: The major enzymatic sources of cytosolic NADPH are indicated, along with their reactants. The sizes of the enzyme boxes are drawn in approximate proportion to their contribution to the cytosolic NADPH pool. The NAD-dependent mitochondrial IDH, which was tested in this study, is also indicated.

The pyridine nucleotides NADH and NADPH are essential co-factors of oxidative and reductive enzymatic processes involved in energetics, oxidative metabolism, redox homeostasis, calcium homeostasis, macromolecular biosynthesis, mitochondrial functions, gene expression, aging and cell death [47]. In this study, we examined the effect of altered levels of the four enzymes that are primarily responsible for reducing NADP to NADPH (Figure 9): two enzymes of the pentose phosphate pathway, G6PD and PGD which together account for ∼40% of NADPH levels in the adult; MEN which generates pyruvate for import into mitochondria and accounts for another ∼30%; and, IDH, which accounts for ∼20% of NADPH [48]–[50]. These enzymes constitute a metabolic network linked by a common substrate (NADP) and interacting regulation [50]. A major finding of our study is that, even though G6PD, PGD, MEN and IDH carry out varied metabolic reactions, alterations to any of their activities have significant consequences for the phenotypes produced by *CryAB^R120G^* expression, implying a common mechanism of action through NADP/H.

In our experiments, reduction of IDH was less effective at *CryAB^R120G^* suppression than reductions of G6PD, PGD or MEN, a result that is not entirely surprising. Alteration of either G6PD or PGD activity is likely to affect both activities coordinately since they constitute sequential steps in the tightly regulated pentose phosphate pathway, and MEN by itself produces more NADPH than any of the other three enzymes of this network. IDH produces less NADPH than either MEN or the G6PD/PGD couple. Additionally, our RNAi-mediated knockdowns of IDH were relatively ineffective. What was surprising was that knockdown of the mitochondrial NAD-dependent IDH resulted in significant suppression of the CryAB^R120G^ phenotype. We surmise that mitochondrial metabolism affects the cytoplasmic NADP/H network.

The NADP∶NADPH redox couple, and the linked glutathione redox couple (GSSG∶GSH), participate in a diverse array of biological processes. Therefore, we envision a number of possible mechanisms through which CryAB^R120G^ could alter the cellular redox potential and thereby contribute to toxicity. Most obviously, redox-sensitive sequestration of both existing and newly synthesized proteins could seriously disturb cellular regulation. The function of many proteins depends on the reduced or oxidized state of thiol-containing cysteine residues. It is conceivable that structurally flexible hydrophobic protein surfaces, which are normally buried within a folded protein, could engage in non-productive protein-protein interactions, in part, due to alterations of intra-chain disulfide links. Partially folded proteins might, in this way, become soluble toxic intermediates [9]. Alternatively, misfolding might occur as a result of alterations in other redox-sensitive post-translational modifications, for example glutathionylation, nitrosylation, and (de)acetylation. Co-aggregation of several distinct polypeptides might cripple multiple disparate functions within the cell. As mentioned, our results also suggest that alterations in mitochondrial homeostasis and energy metabolism could affect the levels of oxidized or reduced NADP/H. The reciprocal is most certainly true as well, with normal mitochondrial function dependent upon the function of the NADP-reducing enzyme MEN. Additionally there are scores of enzymes that use NADP/H as a cofactor, and the activity of one or more of these enzymes could be affected to generate the phenotypes we observed. It will require significant further work to identify the critical determinants of NADP/H involvement in CryAB^R120G^ pathology. *Drosophila* provides powerful tools for genetic screening to identify such factors and the model for *CryAB^R120G^* pathology that we describe here provides a context for carrying out such screens. We anticipate that such efforts may ultimately lead to the identification of potential targets for therapy and the promise of useful treatments for the human disorders.

## Materials and Methods

### Construction of transgenic flies

*P*{*UASP*-*CryAB^+^*} and *P*{*UASP*-*CryAB^R120G^*}: A fragment DNA containing human *CryAB* cDNA was released from donor plasmids (pCMVHA-wtCryAB [51] or pCDH1-MCS1-R120GhCryAB (unpublished); by *Nco*I and *Dra*I digestion, followed by Klenow filling in to make blunt ends. The vector *pUASP* [52] was digested with *Eco*RI or *Not*I respectively and filled in to make blunt ends. Ligation of respective inserts and vectors produced *P*{*UASP*-*CryAB^+^*} and *P*{*UASP*-*CryAB^R120G^*}. Each was verified by restriction enzyme mapping and sequencing, then was used in *P*-element mediated transformation by standard methods [53], [54].

*P*{*UASTattB*-*GFP-CryAB* ^R120G}^: The *CryAB^R120G^* cDNA was released from pCDH1-MCS1-R120GhCryAB by *Eco*RI digestion and was ligated in frame to the same site at the C terminus of *GFP* coding region an intermediate vector. The fusion protein was released using flanking *Xba*I sites and sub-cloned into *pUASTattB* vector provided by J. Bischof [55]. The plasmid was injected into *y w P{ry^+t7.2^, hsFLP}1; M{3xP3-RFP.attP}ZH-86Fb; M{vas-int.B}ZH-102D* (from the Bloomington Drosophila stock center) to integrate the fusion protein construct onto chromosome 3R cytological location 86F. The *X* chromosome carrying *FLP* was removed by crossing after transformation.

### Mapping of transformed *P* elements

Insertion sites of transformed *P* elements were mapped either by inverse PCR or Splinkerette PCR method [56].

### Other *Drosophila* strains and culture conditions

*P*{*tub-GAL4*}, *P*{*ey-GAL4*} and *P*{*Act5C(FRT.y^+^)Gal4*} flies were obtained from the Bloomington, IN, USA, *Drosophila* stock center (lines #5138, #5535 and #25374, respectively). After being exposed to FLP, *P*{*Act5C(FRT.y^+^)Gal4*} lost the *y^+^* marker and became recessive lethal. It was then kept as the balanced stock *y w; P*{*Act5C-GAL4*}/*S^2^ CyO*. *P*{*GMR-GAL4*} flies are discussed in [57]. Fly lines carrying *P*{*UAST-G6PD*} along with control *y w^2^* were provided by W. C. Orr [38]. Mutations for *Zw* and *Pgd* were obtained from Bloomington stock center. RNAi fly stocks were from Vienna *Drosophila* RNAi Center [58]. Table S1 lists the stock numbers of all UAS-RNAi lines (Vienna *Drosophila* RNAi Center), *Zw* overexpression lines (W. C. Orr), and mutants (Bloomington *Drosophila* Stock Center). Flies were raised at 25°C, unless otherwise specified, on standard cornmeal-agar medium in standard 25×90 mm vials.

### Western blotting

Assay for CryAB^R120G^ protein expression: The *UASP*-*CryAB^R120G^* lines were crossed to flies carrying the *tub-GAL4* driver. One male and one female were taken from seven crosses with surviving progeny. Each fly was homogenized in 100 µl of 1X sample buffer. Eight µl of lysate was loaded in each lane of a 12% SDS-PAGE gel, separated by electrophoresis and then examined by Western blotting to detect the CryAB protein. A lysate of mammalian cells expressing *CryAB^R120G^* was included as a positive control. Rabbit antiserum (1∶5000 dilution) against human CryAB protein was the primary antibody [7].

Assay for CryAB protein solubility: To determine whether CryAB expressed in eyes existed in a soluble or insoluble form we modified the procedure described by Carbone *et al.* [59]. Ten heads from females expressing *CryAB* (wildtype or mutant) were collected and homogenized for 10 minutes on ice in 100 µl lysis buffer (10 mM Tris pH 7.5, 5 mM EDTA, 1% NP40, 0.5% deoxycholate, 150 mM NaCl and 1% Triton X-100). After incubation in lysis buffer for 30 minutes on ice, samples were frozen at −20°C overnight, then thawed out. Cuticle and debris in the lysate were separated by brief centrifugation at 1,000 rpm for 1 minute. Supernatant was collected. Soluble and insoluble fractions were further separated by centrifugation at 14,000 rpm for 15 minutes. After collecting the soluble fraction, the insoluble fraction was washed three times with 200 µl lysis buffer each, and then solubilized in 40 µl 1X sample buffer for Western. The soluble fraction was TCA precipitated and washed before being resuspended in 40 µl 1X sample buffer. 2.5 head's worth of soluble or insoluble fraction (10 µl) was loaded into each lane.

Western blotting was carried out following standard procedures using the Odyssey Western Blot Kit (Li-cor). Rabbit antiserum against Human CryAB and mouse anti-β-tubulin (Developmental Studies Hybridoma Bank clone E7) were used as primary antibodies. After incubation with fluorescent anti-rabbit and anti-mouse secondary antibodies, the membrane was scanned on an infrared Odyssey scanner by Li-cor. The Western signal was quantified on the Li-cor scanner and the results from four independent experiments were averaged. The insoluble CryAB is reported as mean percent of total CryAB ± standard error.

### Scoring eye phenotypes

Stocks that carried the *GMR-Gal4* driver and *UAS-CryAB* elements in homozygous condition were generated. Males from this stock were crossed to females from control lines (*y w^2^* or *w^1118^*), or from lines carrying modifying elements or mutations. The eyes of daughters from these crosses were scored for the severity of the eye phenotype by assigning the eye to one of five categories (Figure 1E). All transgenes were hemizygous in the scored females. Mann-Whitney tests for significance were performed using GraphPad Prism software.

### Quantitative RT-PCR

Total RNA from 15–25 female fly heads was harvested using Tri reagent and protocol (Sigma-Aldrich). cDNA was synthesized from total RNA using RevertAid First Strand cDNA Synthesis Kit (Fermentas). Quantitative PCR of the cDNA was carried out on an iQ-PCR machine (Bio-Rad) using Maxima SYBR green/Fluorescein qPCR Master Mix (Fermentas). Relative copy number was calculated against a set of common standard templates for each PCR reaction. For each cDNA sample, the relative copy numbers of gene of interest (X) and ribosomal protein L32 (RPL) were both obtained. Abundance of X in the sample was calculated by dividing the copy number of X by that of RPL. The average of three independent experiments was used to represent the abundance of X in a given genotype.

### *Drosophila* cardiac performance analysis

Two independent wildtype *UAS-CryAB^+^* controls and two *UAS-CryAB^R120G^* mutant fly lines, as well as *UAS-CryAB^R120G^* combined with *UAS-Zw* or *UAS-Zw* RNAi were crossed to *Hand-Gal4* (II) driver flies (*Hand* is a direct target of *Tinman* and *GATA* factors during *Drosophila* cardiogenesis and hematopoiesis, [60]). As an additional control, *Hand-Gal4* (II) driver-flies were crossed to *w^1118^* flies. The progeny were raised at 25°C on standard cornmeal-agar medium. All flies were transferred to fresh food every 2–3 days. At three weeks of age, 45–50 female offspring from each cross were anaesthetized and dissected. All procedures were done at room temperature (18–22°C) as previously described [17], [24], [32], [61], [62]. Briefly, each head, ventral thorax and ventral abdominal cuticle was removed exposing the abdomen [62]. All internal organs and abdominal fat were removed leaving only the heart and associated muscles for each fly. Dissections were performed in oxygenated adult hemolymph. The semi-intact preparations were allowed to equilibrate with oxygenation for 20–30 min prior to filming. Analysis of heart morphology and physiology was performed using high speed movies of the semi-intact *Drosophila* preparations. 30 sec. movies were taken at rates of 100–200 frames/sec. using a Hamamatsu EM-CCD digital camera on a Leica DM LFSA microscope with a 10× immersion lens. All images were acquired and contrast enhanced using Simple PCI imaging software (Compix, Inc.). M-modes were generated and determination of cardiac parameters, including heart periods, diastolic and systolic diameters, fractional shortening and arrhythmicity indices for each group was performed using a MatLab-based image analysis program [63]. The “arrhythmicity index”, which is defined as the standard deviation of the heart period normalized to the median of each fly allowed us to quantify the average severity of arrhythmic beating patterns for each line. One-way ANOVAs of genotype as a function of each measured cardiac parameter, with Bonferroni multiple comparison tests, were employed to determine if significant differences among all *Drosophila* lines were present. *P* values<0.05 were considered significant.

### Fluorescent Imaging of *Drosophila* cardiac tubes

Fluorescent imaging of *Drosophila* heart tubes were performed according to [64]. The *UAS-GFP-CryAB^R120G^* fly line was crossed to *Hand-Gal4* (II) driver flies. The progeny were aged to 1 and 3 weeks. Beating hearts of semi-intact *Drosophila* were placed in artificial *Drosophila* hemolymph containing 10 mM EGTA. Cardiac tubes were examined to ensure contractions were inhibited. Hearts were fixed in 1×PBS containing 4% formaldehyde at room temperature for 20 minutes with gentle shaking. Washing of hearts was performed three times for ten minutes with PBSTx (PBS containing 0.1% Triton-X-100) at room temperature with continual shaking. After washing, the hearts were incubated with Alexa584-phalloidin in PBSTx (1∶1000) for 20 minutes with continual agitation. Washing of the hearts was again carried out three times for ten minutes with PBSTx at room temperature. The hearts were rinsed in 100 µl of PBS for 10 minutes. The specimens were mounted on microscope slides and viewed at 10–25× magnification using a Zeiss Imager Z1 fluorescent microscope equipped with an Apotome sliding module.

### Glutathione assay of adult fly heads

Female flies from transgenic or control lines were crossed to *w^1118^; GMR-Gal4* males. 30–60 heads were collected from flies of the appropriate genotype for each cross and then immediately put in 60–120 µl (2 µl per head) 5% SSA (5-sulfosalicylic acid; Sigma #S-7408) and homogenized with a small eppendorf dounce pestle. Samples were then frozen at −80° and maintained frozen until assayed as described [65].

### Larval glutathione assay

Female flies from *UAS-RNAi*, *UAS-G6PD* or control stocks were collected and crossed to *y w; tub-Gal4/T(2;3)SM6, Cy; TM6,Tb* males. Resulting third instar Tb^+^ larvae were collected and washed with 0.7% NaCl solution. Each collection was weighed and frozen on dry ice. The collected larvae were then stored at −80°C until ready for testing. At least 30 mg of larvae from the same genotype were pooled for each sample. Larvae were homogenized in 1X GSH MES buffer from the Glutathione Assay Kit by Cayman Chemical Company (10 µl/mg sample). The protocol provided with the kit was followed. Total GSH and GSSG measurements were calculated based on the Kinetic Method with minimal time course of 30 minutes.

### Data processing

At least three independent samples were assayed for each genotype. The GSH/GSSG ratio for each sample was calculated independently. Statistical analysis of GSH/GSSG ratios was conducted using Graphpad Prism.

## Supporting Information

Figure S1 Human CryAB^R120G^-GFP fusion protein discretely associates with sarcomeric components of *Drosophila* cardiomyocytes. Cardiac-specific expression from *Hand-Gal4*(II)>*UAS-CryAB^R120G^-GFP* flies results in fluorescently labeled and repetitive myofibrillar components of *Drosophila* cardiac fibers. As found in higher organisms, co-localization of GFP with α-actinin antibodies (not shown) suggests human CryAB likely associates with *Drosophila* Z-discs.
(TIF)

Figure S2 Physiological analysis of cardiac tubes from multiple *UAS*-controlled wildtype (9A and 29A) and mutant (14A and 16A) *CryAB* lines, and of mutant *CryAB* lines with *Zw* overexpression (+G6PD) or RNAi-mediated knockdown (+G6PDRNAi). The data suggest that CryAB^R120G^ is deleterious to several indices of cardiac performance in the two independent mutant fly lines relative to non-mutant CryAB controls. Also, overexpression of *Zw* does not appear to exacerbate the mutant phenotype while *Zw* knockdown substantially improves it. Below the column graphs of each analyzed cardiac parameter are tables summarizing the results of a one way ANOVA followed by a Bonferroni's Multiple Comparison Test. Only comparisons with differences that reach statistical significance are shown. * P≤0.05, ** P≤0.01, *** P≤0.001, **** P≤0.0001.
(TIF)

Figure S3 *UAS-CryAB^R120G^* expression: Western blot of viable and semi-viable transgenic lines carrying *tubGal4* and *UAS-CryAB^R120G^* transgenes. Only the semi-viable line *13A* exhibited any expression, which was seen in males and females. The positive control (left lane) was a cell lysate of mammalian cells that expressed *CryAB^R120G^*. Numbers beneath each lane indicate independent transformed lines.
(TIF)

Figure S4 Suppression of *CryAB^R120G^*-*16A* eye phenotype by co-expression from *CryAB^R120G^* lines *7B* and *13A*. Flies that carried *GMR-Gal4*, *CryAB^R120G^-16A*, and either *CryAB^R120G^-7B* or *CryAB^R120G^-13A* were scored for eye phenotype. Both combinations showed a significant reduction in the severity of the eye phenotype compared to flies carrying only *GMR-Gal4* and *CryAB^R120G^-16A* (Figure 5A).
(TIF)

Table S1 Lines used to modify the levels of NADPH-generating enzymes. Relative RNA levels are given for each line. The stock numbers for various lines used are given. RNAi lines came from the Vienna Drosophila RNAi Center, Vienna, Austria; *Zw* overexpression lines were obtained from W. C. Orr, Dept. of Biological Sciences, Southern Methodist University, Dallas, TX, USA; mutant lines came from the Bloomington Drosophila Stock Center, Bloomington, IN, USA. All lines were tested in hemizygous (transgenes) or heterozygous (mutants) condition in females.
(DOC)

Table S2 Phenotypes produced by *UAS-CryAB^R120G^* lines. All elements were single copy in the flies assayed.
(DOC)
