# Supplementary figures and images for: The NADPH Metabolic Network Regulates Human αB-crystallin Cardiomyopathy and Reductive Stress in Drosophila melanogaster
Source: PLoS Genet. 2013 Jun 20;9(6):e1003544. doi: 10.1371/journal.pgen.1003544 (PMC3688542; doi:10.1371/journal.pgen.1003544)

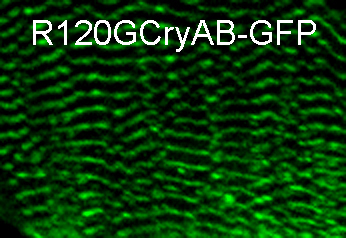

Supplement: Figure S1 — Human CryABR120G-GFP fusion protein discretely associates with sarcomeric components of Drosophila cardiomyocytes. Cardiac-specific expression from Hand-Gal4(II)>UAS-CryABR120G-GFP flies results in fluorescently labeled and repetitive myofibrillar components of Drosophila cardiac fibers. As found in higher organisms, co-localization of GFP with α-actinin antibodies (not shown) suggests human CryAB likely associates with Drosophila Z-discs. (TIF) [file pgen.1003544.s001.tif]

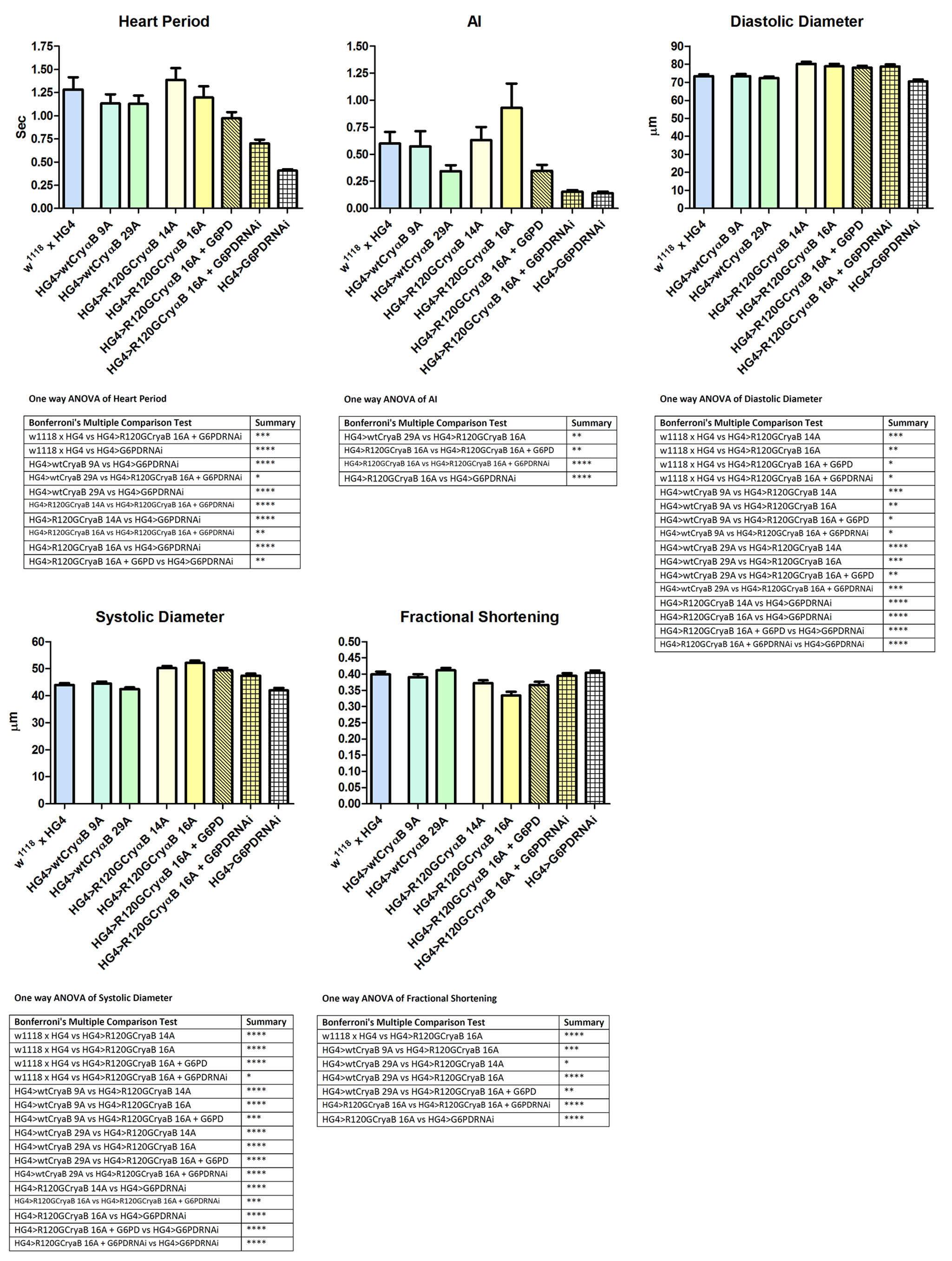

Supplement: Figure S2 — Physiological analysis of cardiac tubes from multiple UAS-controlled wildtype (9A and 29A) and mutant (14A and 16A) CryAB lines, and of mutant CryAB lines with Zw overexpression (+G6PD) or RNAi-mediated knockdown (+G6PDRNAi). The data suggest that CryABR120G is deleterious to several indices of cardiac performance in the two independent mutant fly lines relative to non-mutant CryAB controls. Also, overexpression of Zw does not appear to exacerbate the mutant phenotype while Zw knockdown substantially improves it. Below the column graphs of each analyzed cardiac parameter are tables summarizing the results of a one way ANOVA followed by a Bonferroni's Multiple Comparison Test. Only comparisons with differences that reach statistical significance are shown. * P≤0.05, ** P≤0.01, *** P≤0.001, **** P≤0.0001. (TIF) [file pgen.1003544.s002.tif]

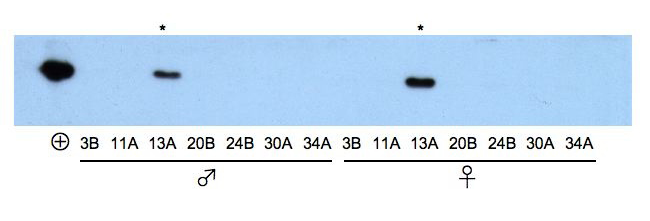

Supplement: Figure S3 — UAS-CryABR120G expression: Western blot of viable and semi-viable transgenic lines carrying tubGal4 and UAS-CryABR120G transgenes. Only the semi-viable line 13A exhibited any expression, which was seen in males and females. The positive control (left lane) was a cell lysate of mammalian cells that expressed CryABR120G. Numbers beneath each lane indicate independent transformed lines. (TIF) [file pgen.1003544.s003.tif]

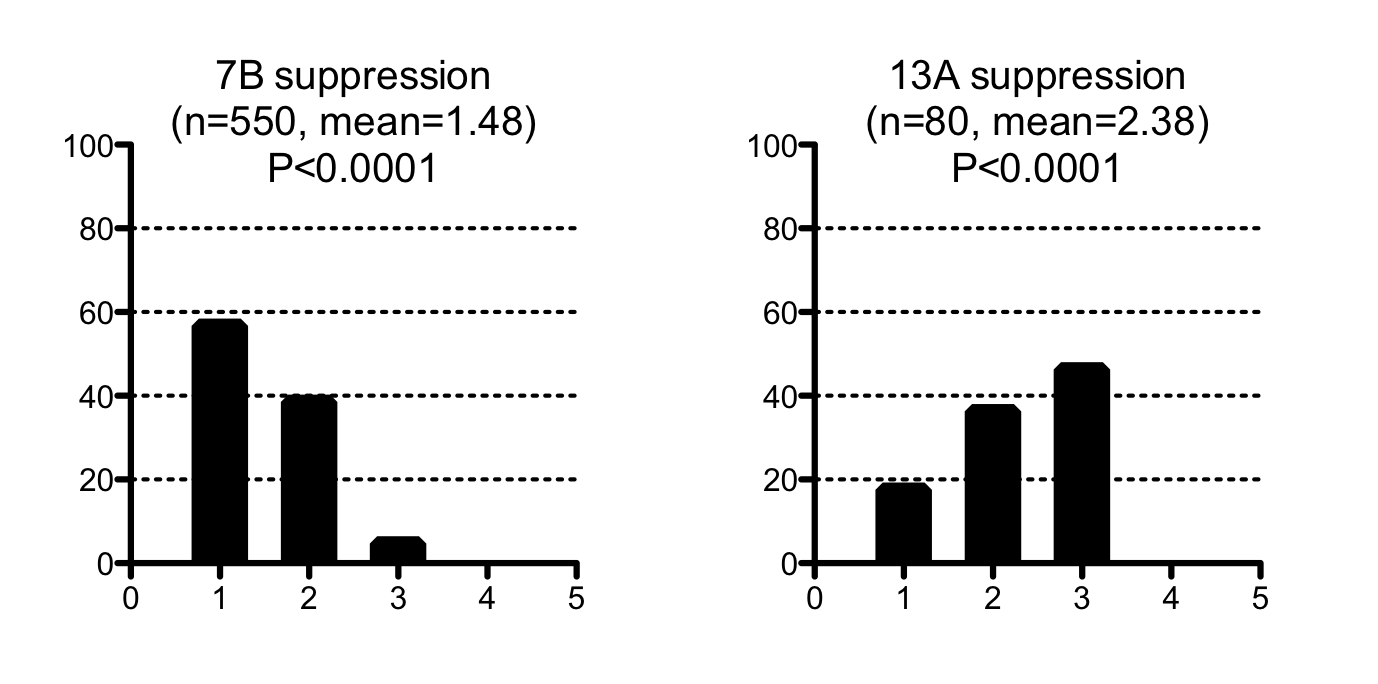

Supplement: Figure S4 — Suppression of CryABR120G-16A eye phenotype by co-expression from CryABR120G lines 7B and 13A. Flies that carried GMR-Gal4, CryABR120G-16A, and either CryABR120G-7B or CryABR120G-13A were scored for eye phenotype. Both combinations showed a significant reduction in the severity of the eye phenotype compared to flies carrying only GMR-Gal4 and CryABR120G-16A (Figure 5A). (TIF) [file pgen.1003544.s004.tif]
