# Supplementary material for: The NADPH Metabolic Network Regulates Human αB-crystallin Cardiomyopathy and Reductive Stress in Drosophila melanogaster
Source: PLoS Genet. 2013 Jun 20;9(6):e1003544. doi: 10.1371/journal.pgen.1003544 (PMC3688542; doi:10.1371/journal.pgen.1003544)
Supplement: Table S1 — Lines used to modify the levels of NADPH-generating enzymes. Relative RNA levels are given for each line. The stock numbers for various lines used are given. RNAi lines came from the Vienna Drosophila RNAi Center, Vienna, Austria; Zw overexpression lines were obtained from W. C. Orr, Dept. of Biological Sciences, Southern Methodist University, Dallas, TX, USA; mutant lines came from the Bloomington Drosophila Stock Center, Bloomington, IN, USA. All lines were tested in hemizygous (transgenes) or heterozygous (mutants) condition in females. (DOC) [file pgen.1003544.s005.doc]

                                     RNA level relative to control

|  | Stock | Figure | *Zw* | *Pgd* | *Men* | *CG6439* | *Idh* (*CG7176*) |
| --- | --- | --- | --- | --- | --- | --- | --- |
| RNAi*^a^* | 3337 | 5B | 0.57 |  |  |  |  |
|  | 101507 | 5C | 0.49 |  |  |  |  |
|  | 100269 | 6E |  | 0.63 |  |  |  |
|  | 104016 | 7G |  |  | 0.61 |  |  |
|  | 14443 | 7C |  |  |  | 0.72 | 0.75 |
|  | 100822 | 7B |  |  |  | 0.52 | 0.77 |
|  | 100554 | 7D |  |  |  |  | 0.71 |
|  | 42916 | 7E |  |  |  |  | 0.88 |
|  | 42915 | 7F |  |  |  |  | 0.82 |
| overexpression*^b^* | 4c | 5E | 162 |  |  |  |  |
|  | 5f | 5F | 164 |  |  |  |  |
|  | 7b | 5G | 145 |  |  |  |  |
|  | 9g | 5H | 253 |  |  |  |  |
| mutation*^c^* | 6033 | 6D | 8.9 | 0.97 |  |  |  |
|  | 7359 | 6B |  | 0.53 |  |  |  |
|  | 2986 | 6C |  | 0.47 |  |  |  |

*^a^*: compared to *w^1118^* control; *^b^*: compared to *y w^2^* control; *^c^*: compared to Canton S control.
