# Supplementary material for: The NADPH Metabolic Network Regulates Human αB-crystallin Cardiomyopathy and Reductive Stress in Drosophila melanogaster
Source: PLoS Genet. 2013 Jun 20;9(6):e1003544. doi: 10.1371/journal.pgen.1003544 (PMC3688542; doi:10.1371/journal.pgen.1003544)
Supplement: Table S2 — Phenotypes produced by UAS-CryABR120G lines. All elements were single copy in the flies assayed. (DOC) [file pgen.1003544.s006.doc]

| R120G lines | *Act-Gal4* | *Tub-Gal4* | *ey-Gal4* | *GMR-Gal4* | *GMR-Gal4* +  Heat shock ^c^ | P{GawB}c179 (BL 6450) | P{GawB}30A  (BL 1795) | *Ser-Gal4*  (BL 6791) |
| --- | --- | --- | --- | --- | --- | --- | --- | --- |
| 16A | lethal ^a^ | lethal ^a^ | Subtle eye phenotype,  some small, irregular eyes | Obvious eye phenotype ^b^ | Enhanced eye phenotype | Female wings folded upwards, male wings crumpled | Female normal, male has wings spread as seen in *Dichaete*. | male lethal, enclosed flies had blisters on the wings |
| 14A | lethal ^a^ | lethal ^a^ | Subtle eye phenotype,  some small, irregular eyes | Obvious eye phenotype ^b^ | lethal | no phenotype | no phenotype | no phenotype |
| 7B | lethal ^a^ | lethal ^a^ | Subtle eye phenotype,  some small, irregular eyes | Subtle eye phenotype | Enhanced eye phenotype, rough eyes | no phenotype | no phenotype | wing vein phenotype at the tip of the wings in both sexes |
| 13A | Semi-lethal ^a^ | Semi-lethal ^a^ | Subtle eye phenotype,  some small, irregular eyes | Subtle eye phenotype | Enhanced eye phenotype, rough eyes | no phenotype | no phenotype | no phenotype |

^a^ not suppressed by *Zw* RNAi

^b^ suppressed by *Zw* RNAi

^c^ 38° 1 hr. heat shock was given at 4-5 days of development.
